# Clinical characteristics and treatment outcomes of women with recurrent uterine leiomyosarcoma

**DOI:** 10.1186/s13023-024-03415-3

**Published:** 2024-10-25

**Authors:** Hua Yuan, Yaxi Wang, Ning Li, Lingying Wu, Hongwen Yao

**Affiliations:** 1 Department of Gynecologic Oncology, National Cancer Center/ National Clinical Research Center for Cancer/ Cancer Hospital, Chinese Academy of Medical Sciences and Peking Union Medical College, 17 # Panjiayuannanli, Chaoyang District, Beijing, 100021 China; 2 Department of Pathology, National Cancer Center/ National Clinical Research Center for Cancer/ Cancer Hospital, Chinese Academy of Medical Sciences and Peking Union Medical College, Beijing, 100021 China

**Keywords:** Recurrent uterine leiomyosarcoma, Clinical characteristics, Treatment, Outcomes

## Abstract

**Objective:**

To determine the clinical characteristics and treatment outcomes of women with recurrent uterine leiomyosarcoma (uLMS).

**Methods:**

We conducted a retrospective cohort study to evaluate the clinical characteristics and survival of women with recurrent uLMS and identify prognostic factors.

**Results:**

Overall, 71 patients with first recurrence of uLMS were included in our study. 19 patients (26.8%) received systemic therapy and 52 patients (73.2%) received secondary cytoreductive surgery (SCS). In SCS subgroup (*n* = 52), a complete resection with no residual disease was performed in 47 patients (90.4%). 38.5% (20/52) patients received non-reproductive organ surgeries. 10 (19.2%) patients had received thoracic surgery because of lung-only recurrence. Bowel, bladder surgery was performed in 8 (15.4%), 3 (5.8%) patients, respectively. 1 (1.9%) patient had received liver surgery. The median follow-up duration was 38.7 months (range: 2.7-317.6 months). 41 (57.7%) patients died during follow-up. 5-year OS for the entire cohort was 52.9%. Patients experienced first recurrence after initial diagnoses within 12 months (*n* = 24) had a worse 5-year OS than those after 12 months (*n* = 47) (17.0% vs. 69.1%, *P* < 0.001). 5-year OS for the SCS and non-SCS subgroup was 62.0% and 28.0%, respectively (*P* < 0.001). Multivariate analysis showed time to fist recurrence within 12 months (HR = 4.60, 95% CI: 1.49–14.4, *P* = 0.008) was an independent predictor of decreased 5-year OS in SCS subgroup.

**Conclusions:**

SCS is an important treatment choice for recurrent uLMS and seems to have benefited patients. Time to fist recurrence within 12 months is an independent predictor of decreased 5-year OS in SCS subgroup.

**Supplementary Information:**

The online version contains supplementary material available at 10.1186/s13023-024-03415-3.

## Introduction

Uterine sarcomas account for approximately 3-7% of all uterine cancers [1]. The most common histologic types of uterine sarcomas are leiomyosarcomas (LMS, 63%), endometrial stromal sarcomas (ESS, 21%), adenosarcomas (6%), undifferentiated sarcoma (5%), and smooth muscle tumors of uncertain malignant potential (STUMP) [2]. Most women with uterine leiomyosarcoma (uLMS) are diagnosed in their 50s and the vast majority present with disease confined to the uterine [3, 4]. Preoperative diagnosis of leiomyosarcoma is difficult and often only made at time of surgical resection. Uterine leiomyosarcoma is an aggressive malignant tumor with a high rate of recurrence [5]. Though the majority (60%) are diagnosed at an early stage, uLMS is still associated with a poor prognosis [6]. The 5-year overall survival rates for stage I, II, III, and IV uLMS were 55.4%, 32.6%, 24.6%, and 13.1%, respectively [7]. Recurrence rate has been reported to be 45–73% in uLMS [8]. Time to first recurrence varies widely and the median intervals are estimated around 12–24 months [9]. Most patients recur within the pelvis and upper abdominal. And the metastasis to the lung is also common.

Very few patients with recurrent or metastatic uLMS can be curatively treated. The prognosis of patients with recurrent or persistent uLMS is poor and the 5-year post-relapse survival rate was 15% [10]. Due to their rarity, the management strategy for patients with recurrent uLMS has not been well established. Treatment choice for recurrent disease is dependent on previous therapy, the site of the recurrent tumor, time to recurrence, and the patient’s performance status [10].

These tumors are relatively chemo and/or radio-resistant. Optimal surgical resection for recurrent uLMS may provide an opportunity for long-term survival in a select patient population [11]. Patients presenting after a prolonged progression-free interval with an isolated site of recurrence amenable to complete resection are the best candidates for attempted surgical resection [8]. Secondary cytoreduction to no residual disease is an option that may be proposed in recurrent uLMS [12]. Modern multimodal therapy or combining chemotherapy with aggressive surgery in selected patients may be significant in prolonging survival of women with this fatal disease [13].

We therefore conducted a retrospective cohort study to evaluate the clinical characteristics and treatment outcomes of women with recurrent uLMS and identify prognostic factors.

## Materials and methods

### Patients

This study was approved by the Research and Ethics Committee of Cancer Hospital, Chinese Academy of Medical Sciences, National Cancer Center. Investigation was conducted in accordance with ethical standards, the Declaration of Helsinki and Chinese and international guidelines. Following Institutional Review Board approval, we performed a retrospective analysis of all patients diagnosed with recurrent uLMS who presented to our institution from January 1, 2001 to January 1, 2020. All patients had previously undergone either total hysterectomy or radical hysterectomy or myomectomy at our center or an outside institution and diagnosed with uLMS after primary surgery which confirmed by two experienced gynecologic pathologists in our hospital.

The diagnostic criteria for uLMS require the presence of the following features: tumour cell necrosis, cytological atypia, or mitoses according to the 2020 WHO Classification of Tumors of Soft Tissue [14]. Immunohistochemically, smooth muscle markers including smooth muscle actin (SMA), desmin, and h-caldesmon are expressed in the majority of uLMSs. Uterine leiomyosarcomas are also immunoreactive for CD10 in a small proportion [15]. Only patients with first recurrent uLMS were included. Patients received treatment in the Department of Gynecological Oncology. Written informed consent was obtained from all patients.

The cohort was divided into two subgroups according to whether receive secondary cytoreductive surgery (SCS) for recurrent uLMS: the SCS subgroup, and non-SCS subgroup. The patients’ full medical records were included in this study. Clinical and pathologic variables, treatment modalities, and outcomes were assessed. Stage was retrospectively assigned using the International Federation of Gynecology and Obstetrics (FIGO) 2008 staging system for uterine sarcomas.

### Statistical analyses

The differences of clinicopathologic characteristics between SCS and non-SCS subgroups were performed using the Pearson χ^2^ test or the Fisher exact test. For the survival analyses, overall survival (OS) was defined as the time from the date of diagnosis to death for which uLMS was the primary or underlying cause. Survival was estimated using the Kaplan–Meier product-limit method, and differences were tested for statistical significance using the log-rank test. Cox proportional hazards regression models were used to identify the prognostic factor [HR and 95% confidence intervals (CI)]. Two-sided P values less than 0.05 were considered to be statistically significant. All analyses were performed using the SPSS Statistics 20.0 software.

## Results

### Patient characteristics

Overall, 71 patients with first recurrent of uLMS were included in our study. Immunohistochemical markers staining results (10X, 20X) of one patient with uLMS were showed in Figure. S1. Patients median age at diagnosis was 48 years (range: 26–69 years). More than half of them were initially diagnosed before 50 years (54.9%). The FIGO 2008 distribution by stage at initial presentation was: stage I in 55 patients (77.5%), stage II in 8 patients (11.3%), stage III in 3 patients (4.2%) and stage IV in 5 patients (7.0%) (Table 1). 51 (71.8%), 17 (23.9%), 3 (4.2%) patients were diagnosed after a total hysterectomy, myomectomy, and radical hysterectomy, respectively (Table 1). Of these patients, 45 (63.4%) received adjuvant chemotherapy, and 3 (4.2%) received adjuvant radiotherapy (Table 1).

**Table 1:** Clinicopathological characteristics of SCS and non-SCS patients in the entire cohort

Clinical Characteristics	All		SCS		Non-SCS		*p*-value
	*n*	%	*n*	%	*n*	%	
**N**	71	100.0	52	73.2	19	26.8	
**Median age (Range)**,** years**	48(26–69)		45.5(26–65)		55(44–69)		0.009
**Age at diagnosis (y)**							0.017
<50	39	54.9	33	63.5	6	31.6	
≥50	32	45.1	19	36.5	13	68.4	
**Parity**							0.198
0	4	5.9	4	8.2	0	0.0	
1	41	60.3	31	63.3	10	52.6	
> 1	23	33.8	14	28.6	9	47.4	
**BMI (kg/m²)**							0.400
≤24	39	54.9	27	51.9	12	63.2	
＞24	32	45.1	25	48.1	7	36.8	
**Pathologic stage (FIGO 2009)**							0.027
I	55	77.5	45	86.5	10	52.6	
II	8	11.3	3	5.8	5	26.3	
III	3	4.2	2	3.8	1	5.3	
IV	5	7.0	2	3.8	3	15.8	
**Surgical route of primary diagnosis**							0.002
Hysterectomy	51	71.8	33	63.5	18	94.7	
Myomectomy	17	23.9	17	32.7	0	0.0	
Radical hysterectomy	3	4.2	2	3.8	1	5.3	
**Initial adjuvant chemotherapy**							0.100
Yes	45	63.4	30	57.7	15	78.9	
No	26	36.6	22	42.3	4	21.1	
**Initial adjuvant radiotherapy**							0.613
Yes	3	4.2	2	3.8	1	5.3	
No	68	95.8	50	96.2	18	94.7	
**Time to fist recurrence**							0.043
≥12 months	47	66.2	38	73.1	9	47.4	
<12 months	24	33.8	14	26.9	10	52.6	
Isolated site*							0.009
Yes	20	28.2	19	36.5	1	5.3	
No	51	71.8	33	63.5	18	94.7	
**Multiple locations** ^**#**^							0.015
Yes	18	25.4	9	17.3	9	47.4	
No	53	74.6	43	82.7	10	52.6	

*Abbreviations* SCS, secondary cytoreduction surgery; non-SCS, non- secondary cytoreduction surgery; BMI, body mass index; FIGO, International Federation of Gynecology and Obstetrics

Notes^*^ Patients who had a recurrence at only 1 site. ^#^ Patients who had a recurrence at 2 or more locations were considered to have multiple locations of recurrence

### Recurrent pattern

The median time from the initial diagnoses to first recurrence was 16.3 months (range: 1.0-161.9 months). 33.8% (24/71) patients experienced recurrence after initial diagnoses within 12 months. And other 66.2% (47/71) patients had first recurrence after 12 months (Table 1).

The most common location of first recurrence was the abdominal/pelvic peritoneum, diagnosed in 47 (66.2%) patients, followed by lung metastases in 24 (33.8%) patients, abdominal wall metastases in 13 (18.3%) patients, bone metastases in 5 (7.0%) patients, vaginal cuff metastases in 6 (8.5%) patients (Table S1).

In the entire cohort, multiple metastases in different locations were found in 18 (25.4%) patients. And 31 (43.7%) patients only had abdominal/pelvic peritoneum recurrence, 16 (22.5%) patients only had lung metastases, 3 (4.2%) patients only had abdominal wall metastases, 2 (2.8%) patients only had vaginal cuff metastases, 1 (1.4%) patient only had bone metastasis (Table 2).

**Table 2:** Recurrent patterns of first relapse disease in the entire population

Recurrent locations	All		SCS		Non-SCS	
	*n*	%	*n*	%	*n*	%
Abdomen/pelvis only	31	43.7	28	53.8	3	15.8
Lung only	16	22.5	10	19.2	6	31.6
Abdominal wall only	3	4.2	3	5.8	0	0.0
Vaginal cuff only	2	2.8	2	3.8	0	0.0
Bone only	1	1.4	0	0.0	1	5.3
Multiple locations^#^	18	25.4	9	17.3	9	47.4

*Abbreviations* SCS, secondary cytoreduction surgery; non-SCS, non- secondary cytoreduction surgery

*Notes*^#^ Patients who had a recurrence at 2 or more locations were considered to have multiple locations of recurrence

### Patient characteristics in different subgroup

In the entire cohort, 19 patients (26.8%) received systemic therapy and 52 patients (73.2%) received SCS. Patients who treated with SCS were younger than those with non-SCS. 63.5% and 31.6% patients were initially diagnosed before 50 years in the SCS and non-SCS subgroup, respectively (*P* = 0.017, Table 1). More patients received myomectomy in SCS subgroup (32.7% vs. 0.0%, *P* = 0.003, Table 1). The majority of patients were assigned to stage I at the time of original diagnosis in SCS subgroup than in non-SCS subgroup (86.5% vs. 52.6%, *P* = 0.008, Table 1). More patients experienced first recurrence after 12 months since diagnosis in SCS subgroup than in non-SCS subgroup (73.1% vs. 47.4%, *P* = 0.043, Table 1).

The recurrent pattern was different in SCS and non-SCS subgroup. Patients treated with SCS were more likely to experience recurrence in isolated sites (36.5% vs. 5.3%, *P* = 0.009, Table 1), and less likely to recur in multiple locations (17.3% vs. 47.4%, *P* = 0.015, Table 1). Patients treated with SCS were more likely to experience recurrence in abdominal/pelvic peritoneum (71.2%), abdominal wall (21.2%) and vaginal cuff (9.6%) (Table S1). Of patients received systemic treatment, 68.4% and 21.1% had lung metastases and bone metastases, respectively (Table S1).

### Secondary cytoreductive surgery treatment

Of the 52 patients undergoing secondary cytoreductive surgery, a complete resection with no residual disease was performed in 47 patients (90.4%) (Table 3). 69.2% patients had a recurrent tumor larger than 5 cm. 38.5% (20/52) patients received non-reproductive organ surgeries. 10 (19.2%) patients had received thoracic surgery because of lung-only recurrence. Bowel, bladder surgery was performed in 15.4%, 5.8% of the cases, respectively. 1 (1.9%) patient had received liver surgery because of liver recurrence. 34.6% patients had estimated blood loss more than 500 ml (Table 3).

**Table 3:** Details of cytoreduction surgery in patients who received SCS

Clinical Characteristics	SCS	
	*n*	%
**Diameter of largest mass found at secondary cytoreduction (cm)**		
<5	16	30.8
≥ 5	36	69.2
**Thoracic surgery**		
Yes	10	19.2
No	42	80.8
**Liver surgery**		
Yes	1	1.9
No	51	98.1
**Bowel resection**		
Yes	8	15.4
No	44	84.6
**Bladder surgery**		
Yes	3	5.8
No	49	94.2
**Residual tumor**		
No	47	90.4
Yes	5	9.6
**Estimated blood loss**		
<500 ml	34	65.4
≥ 500 ml	18	34.6
**Adjuvant therapy after SCS**		
None	16	30.8
Target therapy	1	1.9
Chemotherapy	34	65.4
Chemotherapy + Radiotherapy	1	1.9%

*Abbreviations* SCS, secondary cytoreduction surgery

### Adjuvant therapy after SCS

35 (67.3%) patients received adjuvant chemotherapy after SCS (Table 3). The most common chemotherapy regimen was doxorubicin-based treatment, followed by gemcitabine/docetaxel regimen. 1 (1.9%) patient with vaginal cuff recurrent received pelvic radiotherapy. 1 (1.9%) patient received pazopanib treatment. 16 (30.8%) patients did not receive any adjuvant therapy after SCS.

### Non-secondary cytoreductive surgery treatment

Among patients received non-secondary cytoreductive surgery treatment, 14 (73.7%) patients received chemotherapy, 2 (10.5%) patients received chemotherapy and pelvic radiotherapy, 1 (5.3%) patient received anlotinib treatment, and 2 (10.5%) patients refused cancer treatment (Table S2). 16 patients died during follow-up.

### Survival analysis

**Fig. 1:**
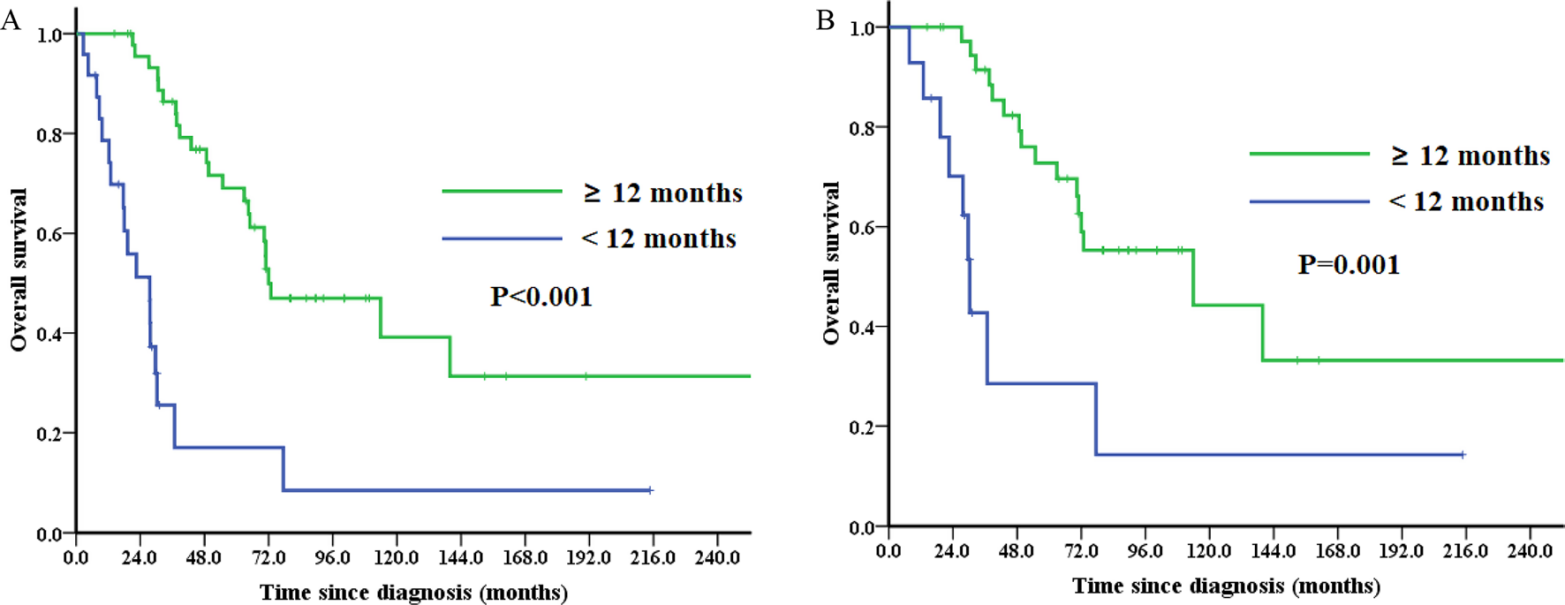
Overall survival (OS) analyses by the Kaplan–Meier method according to the time to first recurrence after initial diagnoses in (**A**) the entire subgroup (*n* = 71) and (**B**) the SCS subgroup (*n* = 52)

**Fig. 2:**
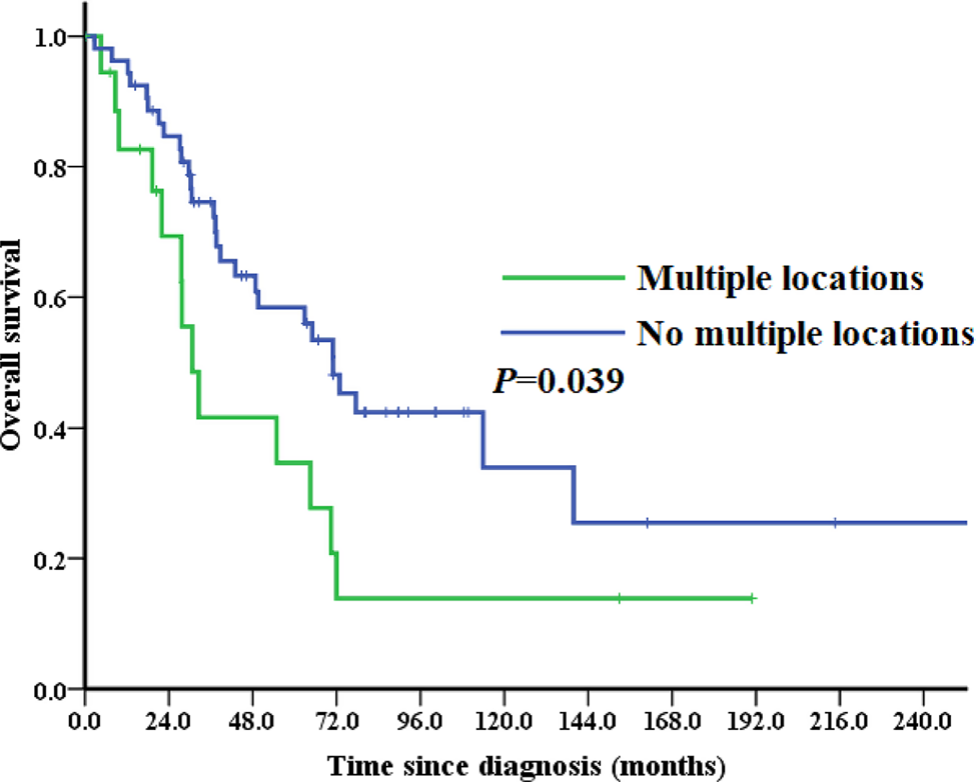
Overall survival (OS) analyses by the Kaplan–Meier method according to whether or not had the multiple locations recurrence in the entire cohort (*n* = 71). Patients who developed recurrence in multiple locations had a significantly worse survival (5-year OS: 58.4 vs. 34.7 *P* = 0.039)

The median follow-up duration was 38.7 months (range: 2.7-317.6 months). 41 (57.7%) patients died during follow-up. 5-year OS for the entire cohort was 52.9% (Figure. S2). Stage-specific 5-year OS were as follows: stage I—60.7%, stage II-IV—27.8% (*P* = 0.001; Figure. S3). Patients experienced first recurrence after initial diagnoses within 12 months had a worse 5-year OS than those after 12 months (17.0% vs. 69.1%, *P* < 0.001, Fig. 1A). 5-year OS for the SCS and non-SCS subgroup was 62.0% and 28.0%, respectively (*P* < 0.001; Figure. S4). Patients who recurred at isolated site associated with a better survival (5-year OS: 73.5% vs. 44.0%, *P* = 0.045; Figure. S5). Patients who developed recurrence in multiple locations had a significantly worse survival (5-year OS: 58.4% vs. 34.7% *P* = 0.039, Fig. 2).

Of the 52 patients undergoing SCS, patients experienced first recurrence after initial diagnoses within 12 months had a worse 5-year OS than those after 12 months (28.5% vs. 72.8%, *P* = 0.001, Fig. 1B). Patients with residual tumors after cytoreductive surgery had a tendency towards a worse survival than those without (5-year OS: 20.0% vs. 67.7%, *P* = 0.082; Figure. S6). Patients who received non-reproductive organ surgeries had a non-significantly worse survival than those who did not receive (5-year OS: 51.5% vs. 69.8%, *P* = 0.057; Figure. S7). And patients with lung-only recurrence (*n* = 10) had a tendency towards a better 5-year OS than those without (*n* = 42) (77.8%% vs. 57.8%, *P* = 0.938; Figure. S8).

Multivariate analysis showed that time to fist recurrence within 12 months (HR = 4.60, 95% CI: 1.49–14.4, *P* = 0.008, Table 4) was an independent predictor of decreased 5-year OS after adjusted time to fist recurrence, diameter of largest mass found at SCS, isolated site recurrence, multiple locations, non-reproductive organ surgeries, residual tumor, adjuvant chemotherapy.

**Table 4:** Univariate and multivariate analyses of OS in SCS subgroup (*n* = 52)

Clinical Characteristics	Univariate analysis of OS			Multivariate analysis of OS		
	HR	95%CI	*P*	HR	95%CI	*P*
**Time to fist recurrence (months)**						
<12 vs ≥ 12	3.91	1.68–9.10	0.001	4.60	1.49–14.41	0.008
**FIGO stage**						
II-IV vs I	2.64	0.98–7.13	0.047	1.48	0.39–5.53	0.563
**Diameter of largest massfound at SCS (cm)**						
≥5 vs < 5	1.23	0.53–2.86	0.629	1.68	0.56–5.04	0.359
Isolated site*						
No vs Yes	1.57	0.69–3.58	0.281	1.13	0.41–3.15	0.813
**Multiple locations** ^#^						
Yes vs No	2.07	0.82–5.21	0.116	4.58	1.42–14.77	0.011
**Non-reproductive organs surgeries**						
Yes vs No	2.12	0.96–4.69	0.057	2.34	0.79–6.97	0.125
**Residual tumor**						
Yes vs No	2.54	0.86–7.53	0.082	2.32	0.52–10.31	0.269
**Adjuvant chemotherapy**						
No vs Yes	1.26	0.52–3.08	0.778	1.27	0.44–3.61	0.659

*Abbreviations* OS, overall survival; HR, hazard ratio; CI, confidence interval; SCS, secondary cytoreduction surgery

*Notes* * Patients who had a recurrence at only 1 site. # Patients who had a recurrence at 2 or more locations were considered to have multiple locations of recurrence

## Discussion

In the present study, the clinical characteristics and treatment outcomes of 71 patients with recurrent uterine leiomyosarcoma treated at our institution were analyzed. To our knowledge, the current study is one of the largest studies to evaluate the clinical characteristics and treatment outcomes of women with recurrent uLMS in a single center to data. We found that secondary cytoreductive surgery is an important treatment choice for recurrent uLMS and time to fist recurrence within 12 months is an independent predictor of decreased 5-year OS in patients who received SCS. These findings suggested that it’s important to identify the suitable candidates for SCS.

Uterine leiomyosarcoma is the most frequent malignant gynecologic mesenchymal tumor, often develops distant metastases and local recurrence [1, 4]. Because of their low incidence and the lack of prospective studies, it is very difficult to reach conclusions as to the best disease management recommendations for recurrent uLMS. Treatment recommendations are made according to the site and nature of the recurrence for recurrent uLMS. Emerging evidence suggested that optimal surgical resection for recurrent uLMS may provide an opportunity for long-term survival in a select patient population [8, 11, 12, 16–19]. The survival advantage was seen not only in patients with pulmonary metastases but also patients with extrapulmonary metastases [8]. In the present study, we found secondary cytoreduction surgery in patients with first recurrent uLMS was associated with a significant improvement in overall survival. Recently, some studies showed cytoreductive surgery and hyperthermic intraperitoneal chemotherapy (CRS/HIPEC) was a promising treatment modality for uterine sarcoma patients with peritoneal dissemination [20–22]. ^6–8^ It’s important to identify the suitable candidate for SCS.

The time to first recurrent since initial diagnosis affects the survival. Patients with uLMS who experience a longer time to recurrence may have improved survival outcomes following metastasectomy [11]. In the present study, patients experienced first recurrence after initial diagnoses within 12 months had a significantly worse 5-year OS than those after 12 months, which was an independent predictor of worse survival in SCS subgroup. The underlying reason may be these patients may have a more aggressive tumor behavior [23]. The choice of treatment method may need to be combined with the timing of the patient’s relapse.

Site governs local control, distant recurrence-free and disease-specific survival for completely resected locally recurrent sarcoma without metastasis [24]. Patients with single site recurrence are more likely to receive SCS and achieve a complete resection with no residual disease than those with multiple sites recurrences. We found patients with multiple recurrent locations were more likely to receive systemic therapy and had a worse survival, which in accordance with other studies. Similarly, patients with residual tumors after cytoreductive surgery had a tendency towards a worse survival than those without in the present study, although the sample size was small.

Emerging evidence suggested that, the most frequent distant metastatic sites for uLMS were lung (67.7%) [9]. We found lung was also the most common distant metastatic site in our study. But it’s not that bad for some patients, especially for those with lung-only recurrence. Our results suggested that patients with lung-only recurrence had a tendency towards a better 5-year OS than those without, although these patients had a hematogenous spread.

uLMS also have a high tendency for local recurrence in pelvic and abdominal cavity after initial treatment. Since it is difficult to discriminate between benign uterine fibroids and uterine sarcomas preoperatively, most uterine sarcomas are often found incidentally after primary hysterectomy or myomectomy [25, 26]. Tumor fragmentation/morcellation might be used which was associated with significantly higher risk of recurrence and a nearly 4-fold increase in peritoneal recurrence [27]. Nearly all patients had received primary myomectomy in other centers, we could not determining how many patients had received morcellation clearly in the present study.

Radiotherapy can be recommended for patients with recurrent uterine sarcoma based on tumor resectability and patients’ prior radiotherapy exposure. For patients with local recurrent, all recurrences are localized either in or directly proximal to the vaginal cuff that is negative for distant metastatic disease. Radiotherapy or surgery treatment are reasonable choices. Concurrent radiotherapy shows good local effectiveness with a good long-term survival for local recurrence [28]. A combined modality approach with perioperative external beam radiotherapy (EBRT), surgery, and intraoperative radiation therapy (IORT) for locally advanced or recurrent uterine sarcoma resulted in excellent local disease control with acceptable toxicity, even in patients with positive resection margins [29]. 8.5% patients had vaginal cuff recurrent in our study and 3 of them received pelvic radiotherapy in our study.

Further adjuvant systemic therapy should be considered for patients with recurrent leiomyosarcoma after initial surgical treatment or radiotherapy. Systemic therapy is also important medical choice for patients with distant metastasis. Leoimyosarcoma is extremely aggressive and responds poorly to traditional chemotherapeutics. Docetaxel/gemcitabine, doxorubicin, and ifosfamide are all reasonable options for advanced or recurrent disease with response rates ranging from 17 to 36% [1, 30]. Gemcitabine and docetaxel have demonstrated the highest objective response rates as first-line or second-line treatment for metastatic disease, with an OS of 14.7 months in second-line treatment [31]. Gemcitabine-docetaxel remains a standard first-line treatment for uLMS [32]. Recently, new drugs such as trabectedin and eribulin have showed promising therapeutic effect for patients with recurrent uLMS [33, 34]. Olaparib and temozolomide also provide meaningful clinical benefit in patients with advanced, pretreated uLMS [35]. Several novel therapeutic targets for uterine leiomyosarcoma have been identified and the preclinical efficacy of novel drug candidates have been assessed [36–40]. The most common chemotherapy regimens for recurrent uLMS were doxorubicin-based regimens and docetaxel/gemcitabine in our study.

Target therapy is important choice for patients with recurrent sarcoma. In recent years, targeted therapies such as pazopanib and olaratumab achieved a highly significant improvement in survival for patients with metastatic uLMS [41, 42]. Larotrectinib is highly active treatment especially for patients with NTRK fusions [43]. The potential role of immunotherapy is being assessed in current uLMS clinical trials. Doxorubicin in combination with pembrolizumab is a promising combination worthy of further study, especially in certain sarcoma subtypes [44, 45]. Endocrine therapy is also an important treatment for recurrent sarcoma. Aromatase inhibitors can be considered for ER/PR-expressing uLMS [46].

There are two limitations to our study. The current study was retrospective, and the primary treatment was not assigned at randomized. All patients with recurrent uLMS in this study came from our single center. Therefore, caution is required when interpreting our results.

## Conclusions

In conclusion, recurrent uLMS are a rare group of tumors with an aggressive behavior and poor outcomes. The current study shows that secondary cytoreductive surgery is an important treatment choice for these patients and seems to have benefited patients. Time to fist recurrence within 12 months is an independent predictor of decreased 5-year OS in SCS subgroup. It’s important to identify the suitable candidate for SCS. A prospective large study is warranted to validate these findings.

## Electronic supplementary material

Below is the link to the electronic supplementary material.

Supplementary Material 1

Supplementary Material 2

## Data Availability

The data are not publicly available due to the containing information that could compromise the privacy of research participants. The datasets generated during and/or analyzed during the current study can be provided upon reasonable request to the corresponding author.
