# Supplementary material for: Clinical characteristics and treatment outcomes of women with recurrent uterine leiomyosarcoma
Source: Orphanet J Rare Dis. 2024 Oct 25;19:395. doi: 10.1186/s13023-024-03415-3 (PMC11515372; doi:10.1186/s13023-024-03415-3)

**Supplementary Figures**

**Figure S1.** Immunohistochemical markers staining results (10X, 20X) of one patient with uterine leiomyosarcoma.

1. HE


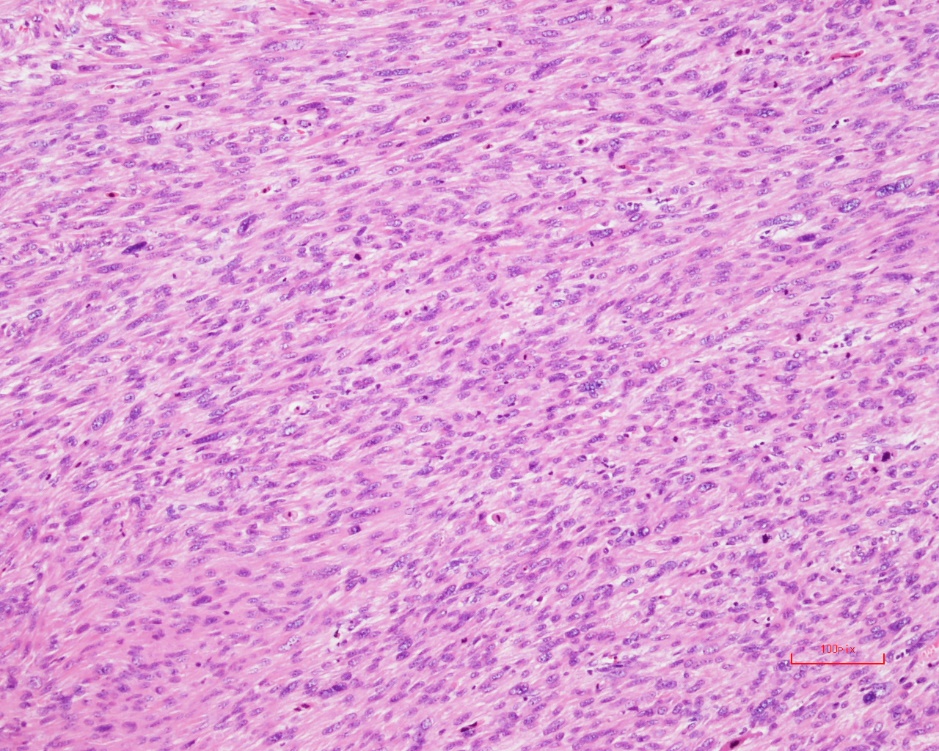


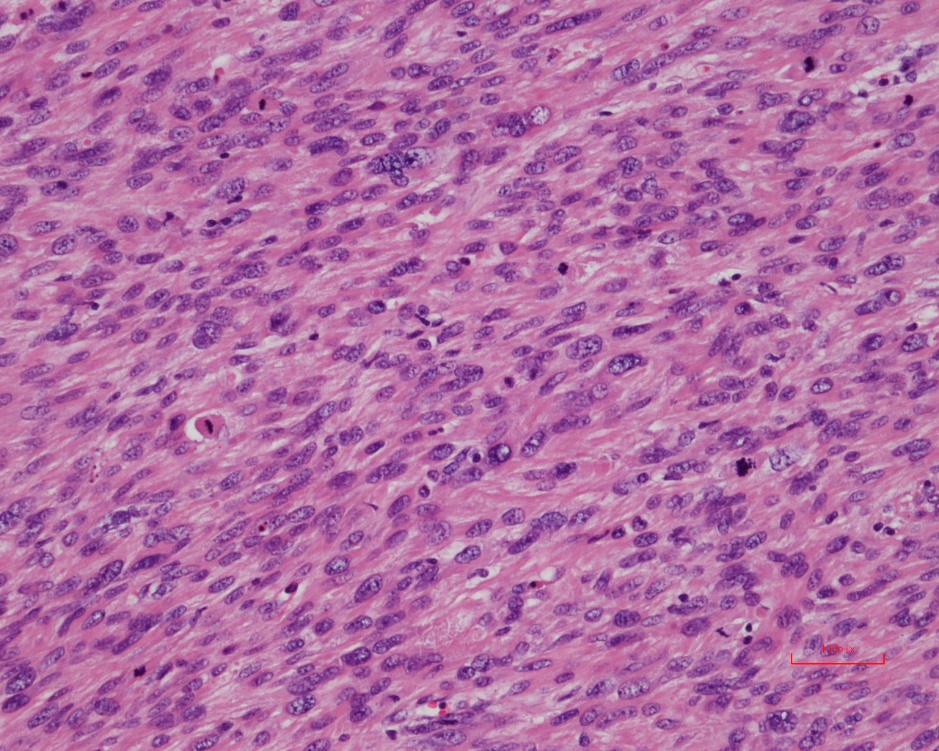


1. SMA (diffuse positive)


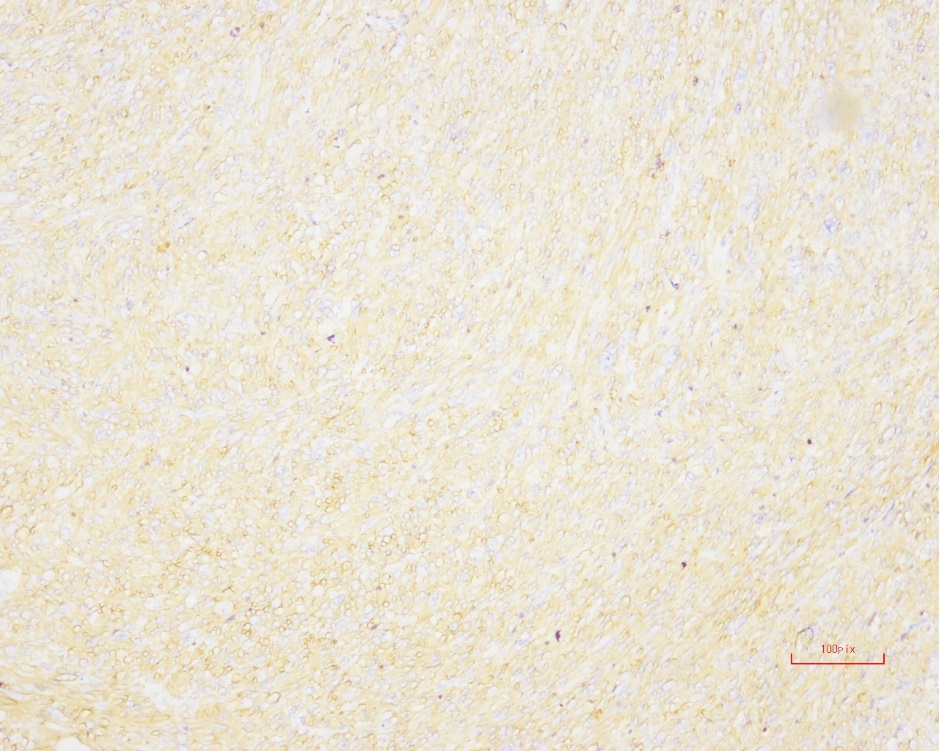


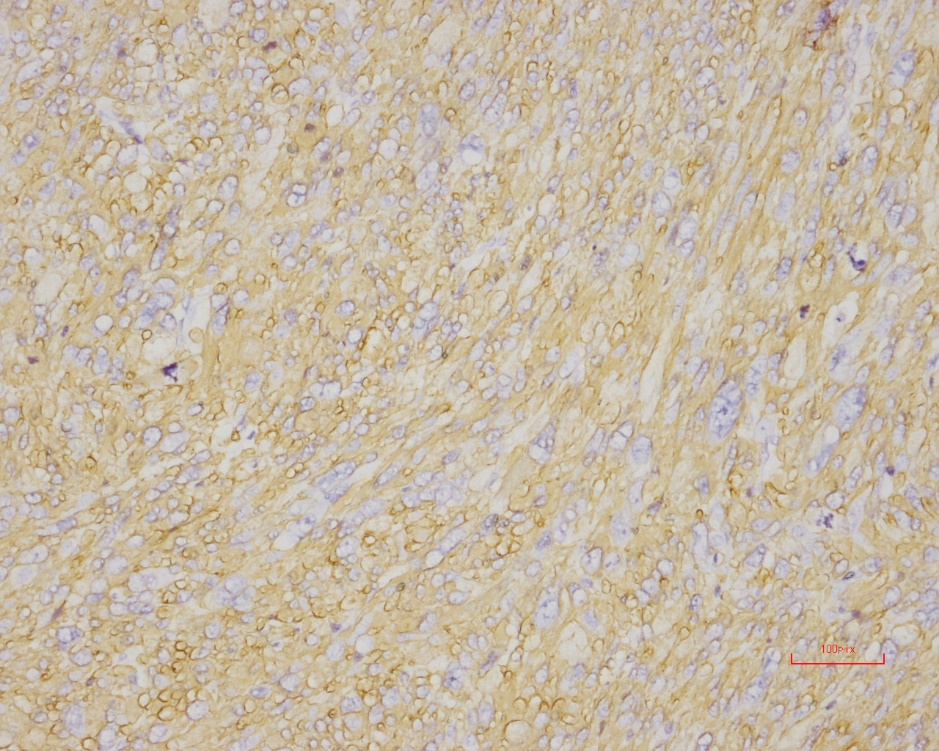


1. Desmin (diffuse positive)


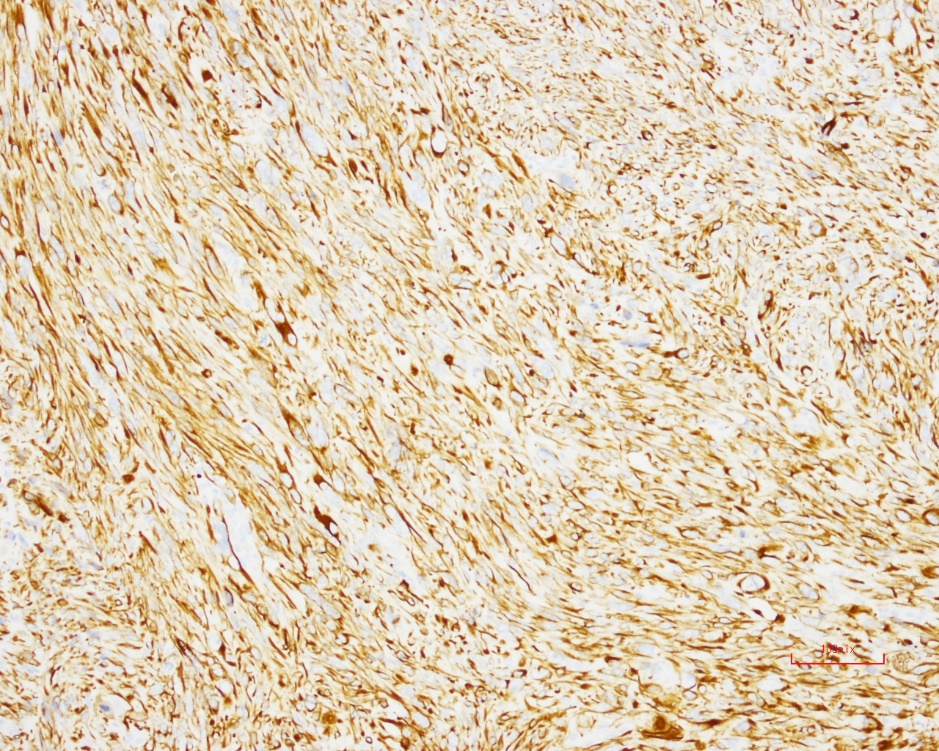


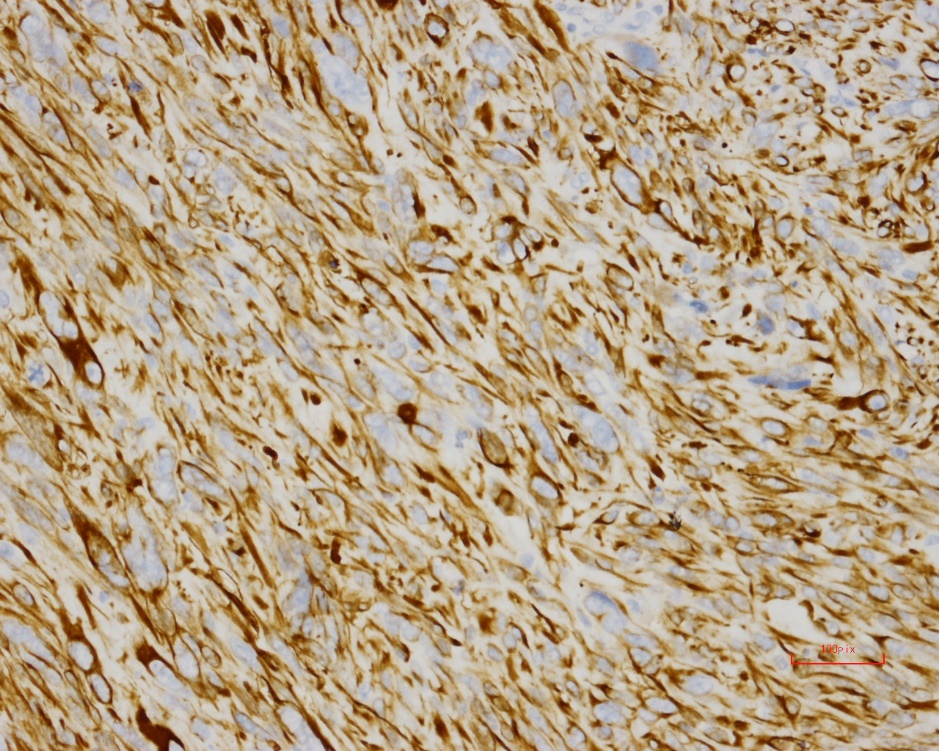


1. Caldesmon (diffuse positive)


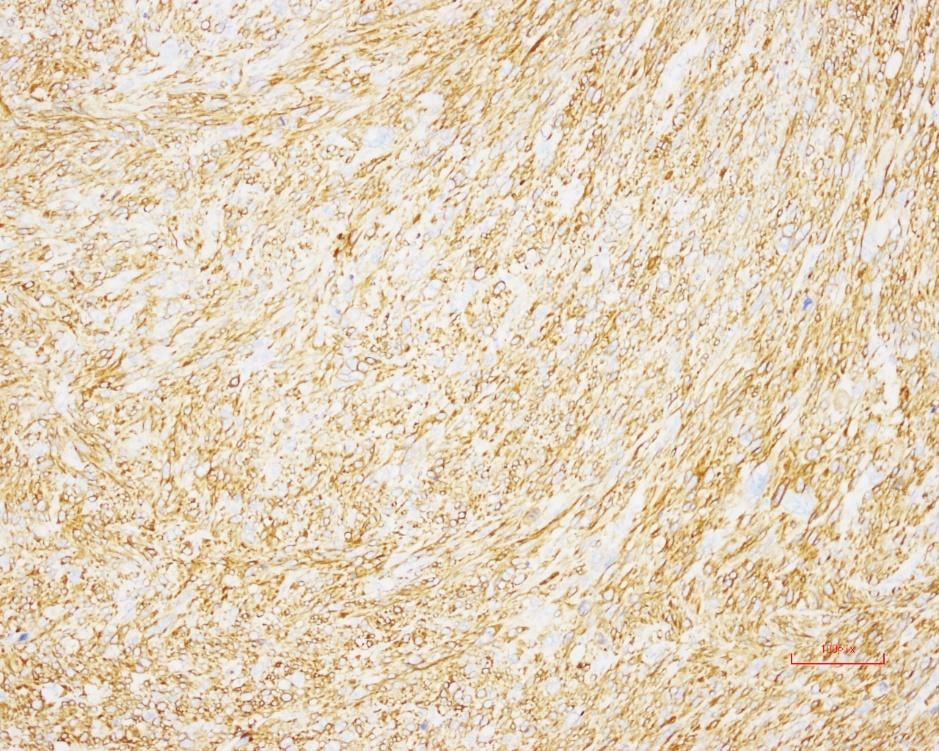


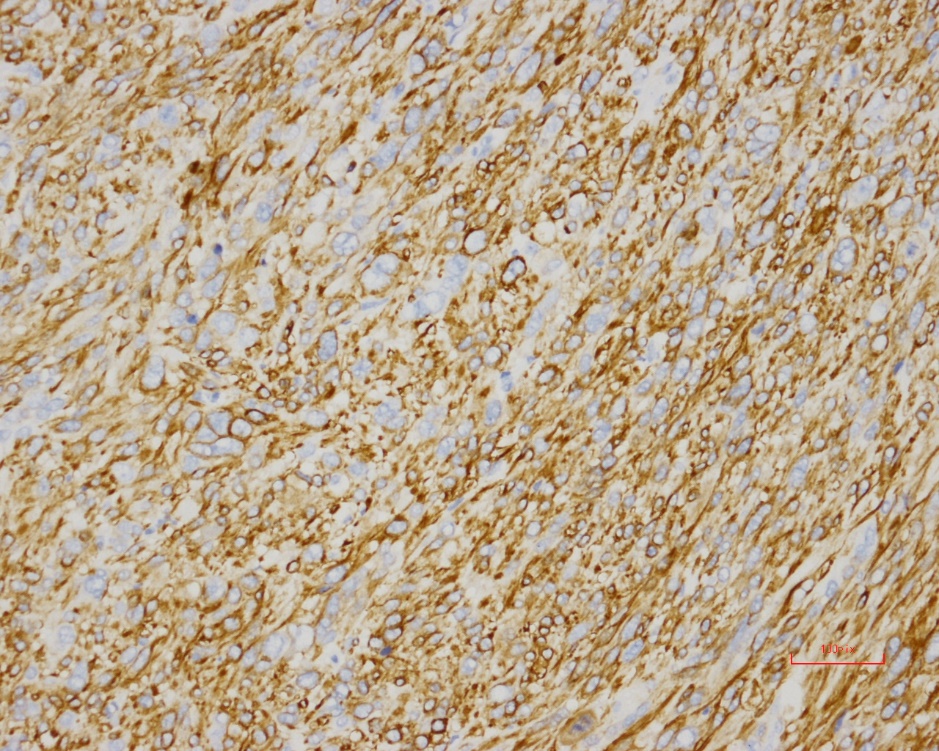


1. CD10 (negative)


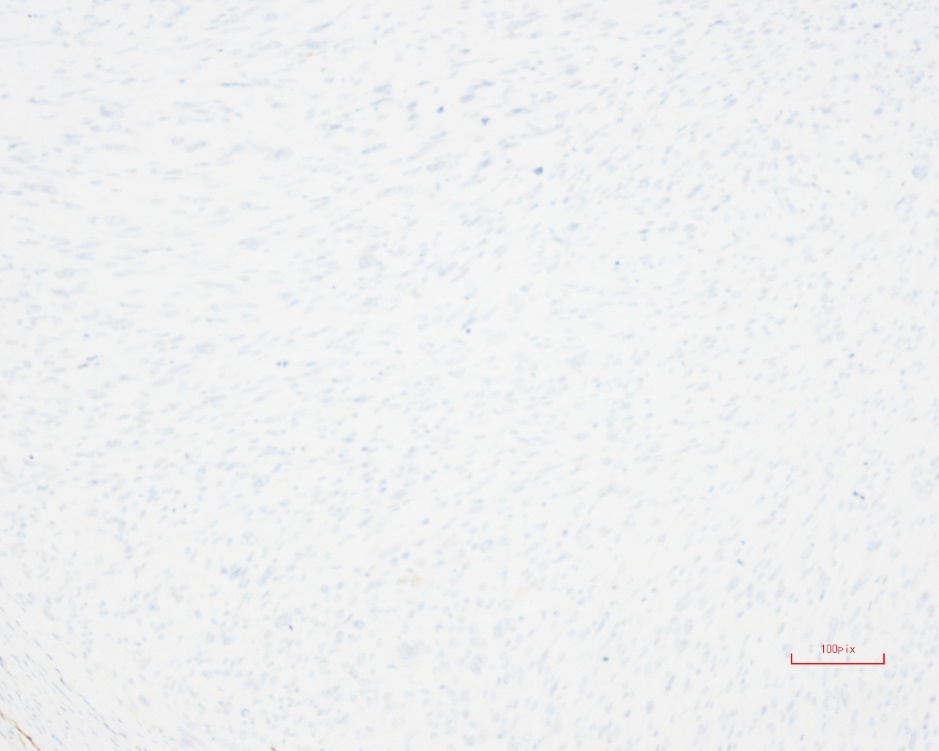


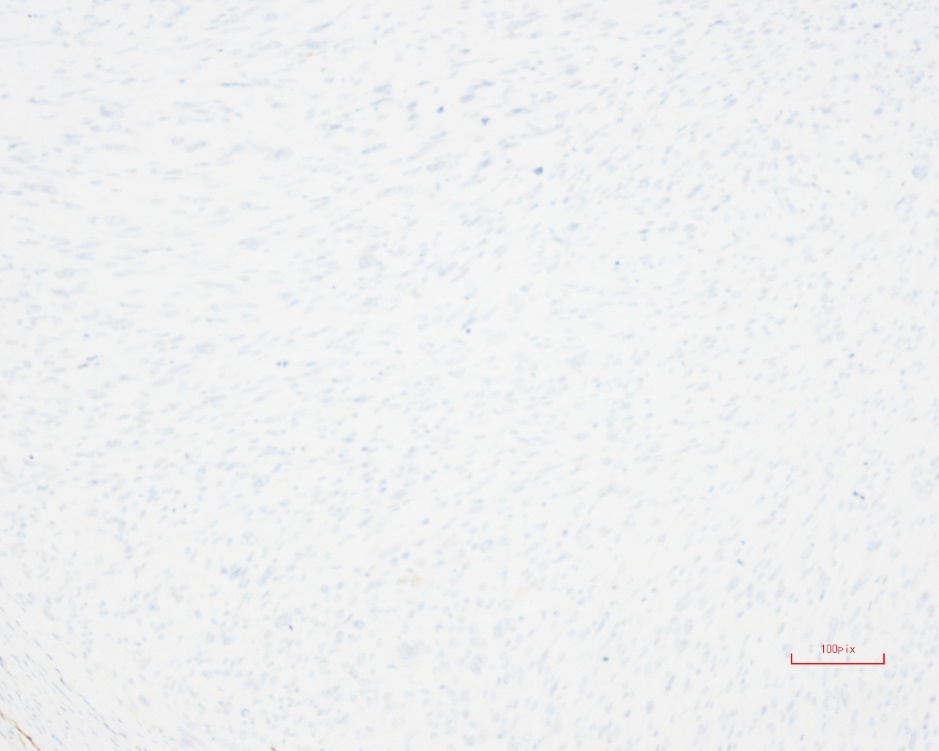


1. Ki67 (+ 70%)


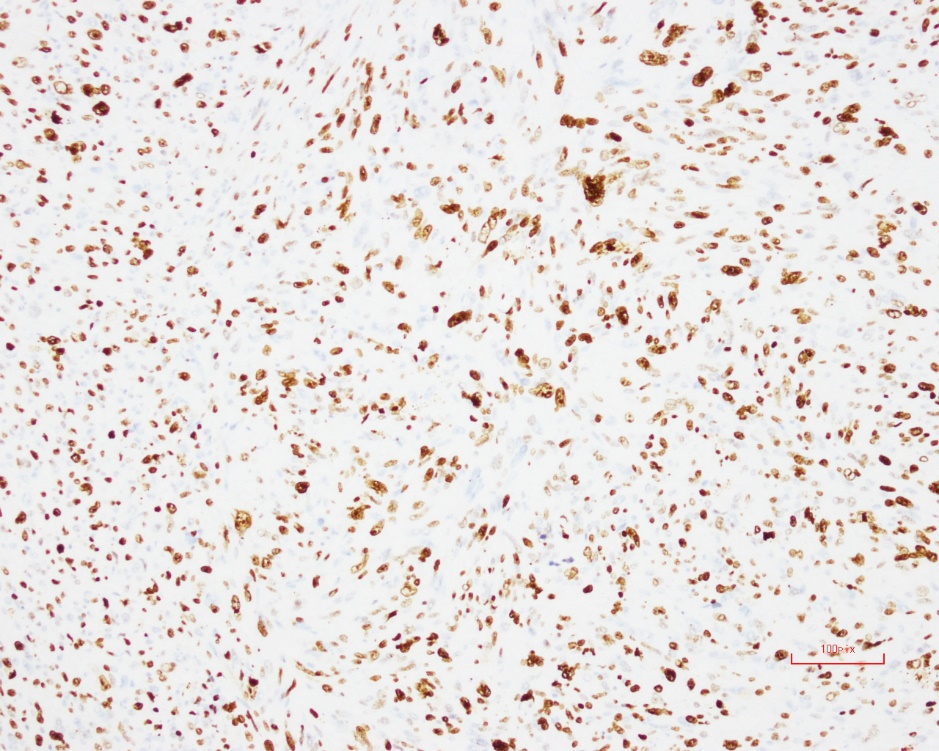


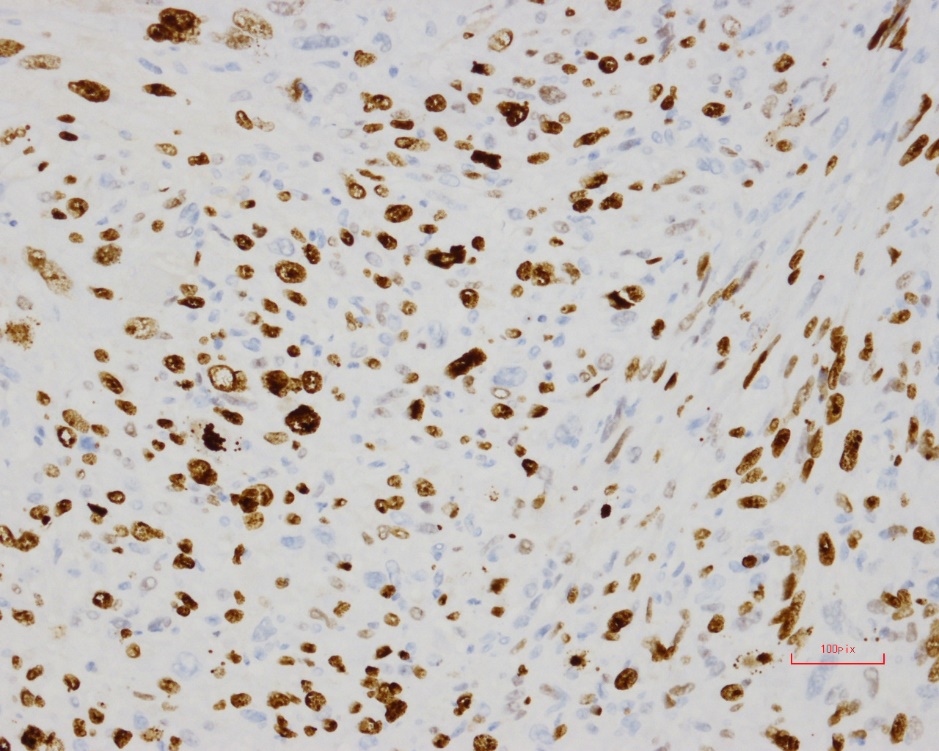


**Figure S2.** Overall survival (OS) analyses by the Kaplan–Meier method in the entire cohort (n=71).


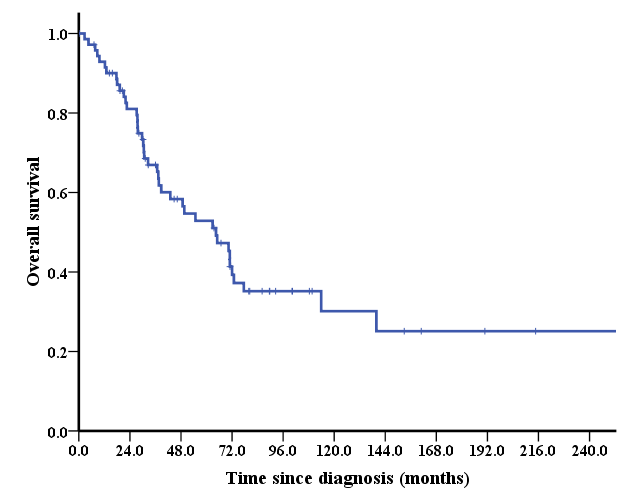


**Figure S3.** Overall survival (OS) analyses by the Kaplan–Meier method according to the tumor stage in the entire cohort (n=71).


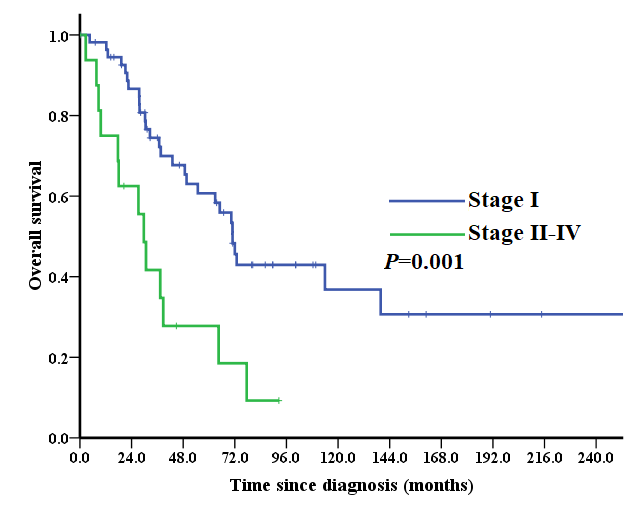


**Figure S4.** Overall survival (OS) analyses by the Kaplan–Meier method according to whether or not received SCS for the first recurrence after initial diagnoses in the entire cohort (n=71).


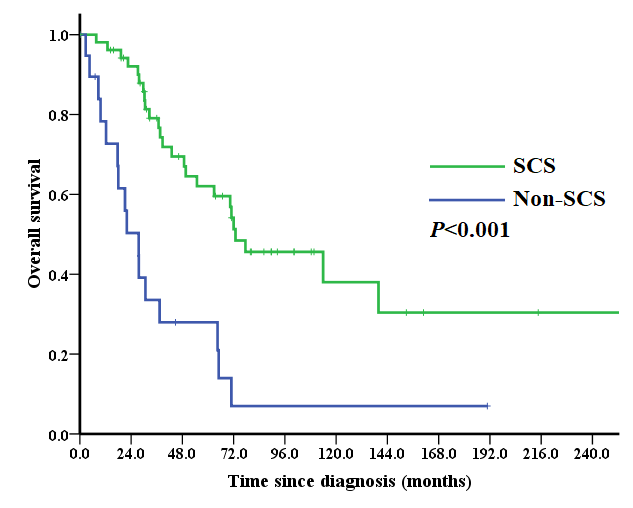


**Figure S5.** Overall survival (OS) analyses by the Kaplan–Meier method according to whether or not had the isolated recurrent site in the entire cohort (n=71).


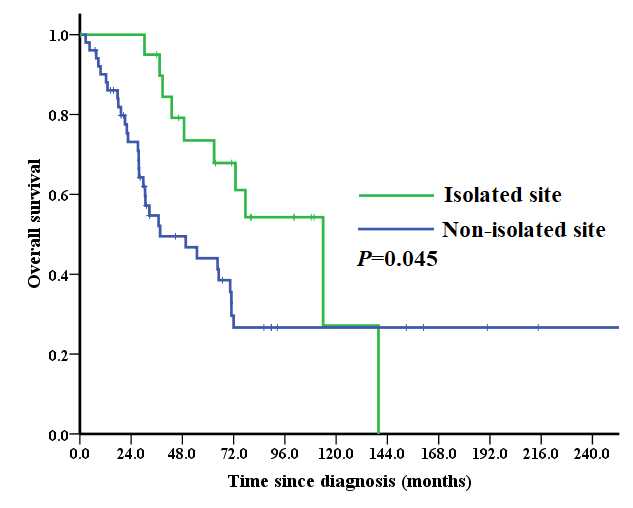


**Figure S6.** Overall survival (OS) analyses by the Kaplan–Meier method according to residual tumors status in the SCS subgroup (n=52).


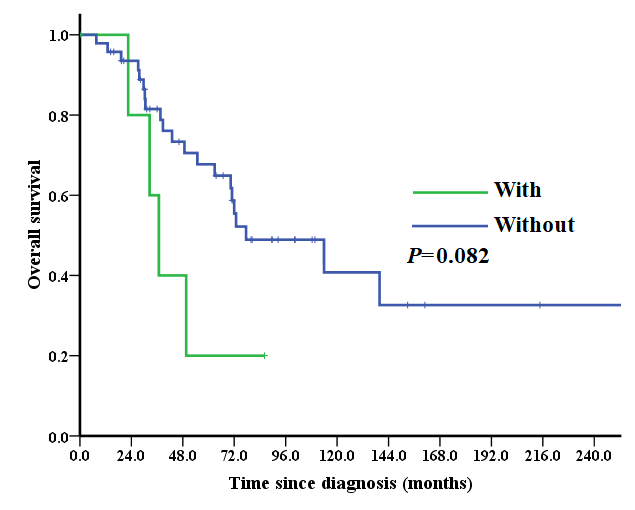


**Figure S7.** Overall survival (OS) analyses by the Kaplan–Meier method according to whether or not had received non-reproductive organ surgeries in the SCS subgroup (n=52).


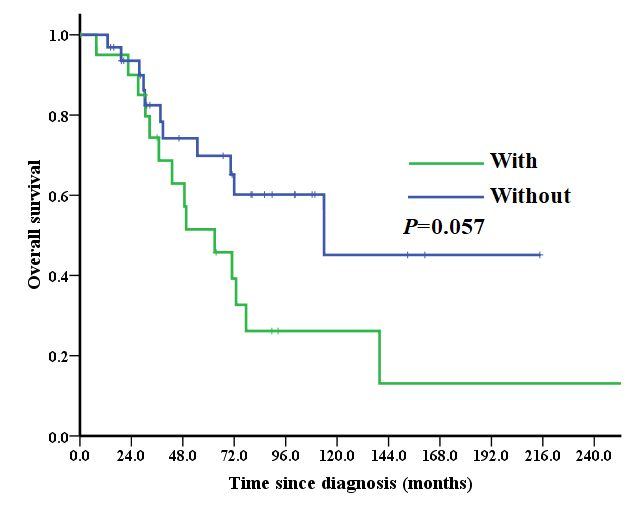


**Figure S8.** Overall survival (OS) analyses by the Kaplan–Meier method according to lung recurrence status in the SCS subgroup (n=52).


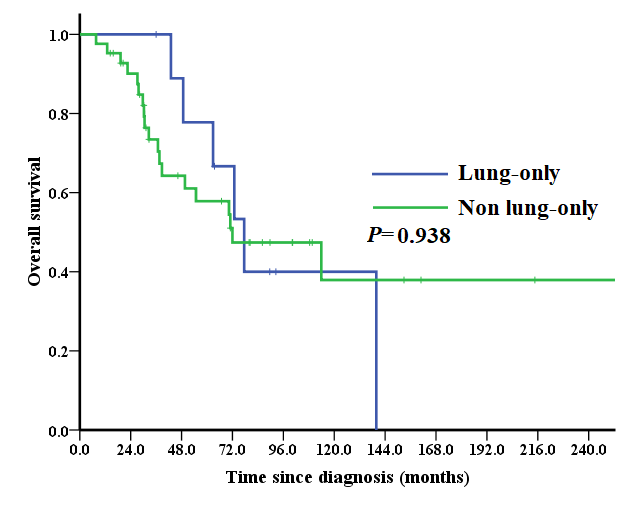

Supplement: Supplementary file 1 — Supplementary Material 1 [file 13023_2024_3415_MOESM1_ESM.docx]
