# Supplementary material for: Clinical characteristics and treatment outcomes of women with recurrent uterine leiomyosarcoma
Source: Orphanet J Rare Dis. 2024 Oct 25;19:395. doi: 10.1186/s13023-024-03415-3 (PMC11515372; doi:10.1186/s13023-024-03415-3)
Supplement: Supplementary file 2 — Supplementary Material 2 [file 13023_2024_3415_MOESM2_ESM.docx]

**Supplementary Tables**

**Table S1.** Locations of the first relapse disease in the entire population

| **Recurrent locations** |  | **All** | |  | **SCS** | |  | **Non-SCS** | |
| --- | --- | --- | --- | --- | --- | --- | --- | --- | --- |
|  |  | **n** | **%** |  | **n** | **%** |  | **n** | **%** |
| Abdomen/pelvis^#^ |  | 47 | 66.2 |  | 37 | 71.2 |  | 10 | 52.6 |
| Lung |  | 24 | 33.8 |  | 11 | 21.2 |  | 13 | 68.4 |
| Abdominal wall |  | 13 | 18.3 |  | 11 | 21.2 |  | 2 | 10.5 |
| Vaginal cuff |  | 6 | 8.5 |  | 5 | 9.6 |  | 1 | 5.3 |
| Bone |  | 5 | 7.0 |  | 1 | 1.9 |  | 4 | 21.1 |

Abbreviations: SCS, secondary cytoreduction surgery; non-SCS, non- secondary cytoreduction surgery.

Notes: ^#^Some patients recurred in 2 or multiple locations, and patients might be included in more than 1 category.

**Table S2.** Treatment for non-SCS patients

| **Treatment** |  | n | % |
| --- | --- | --- | --- |
| None |  | 2 | 10.5 |
| Chemotherapy |  | 14 | 73.7 |
| Chemotherapy+ Radiotherapy | | 2 | 10.5 |
| Target therapy |  | 1 | 5.3 |

Abbreviations: Non-SCS, non secondary cytoreduction surgery.
